# Prise en charge des traumatismes graves du rein

**DOI:** 10.11604/pamj.2015.20.116.1107

**Published:** 2015-02-10

**Authors:** Mohamed Amine Lakmichi, Redouane Jarir, Bader Sadiki, Zehraoui, Bentani, Bader Wakrim, Zakaria Dahami, Moudouni, Ismail Sarf

**Affiliations:** 1 Service d'Urologie, Faculté de Médecine et de Pharmacie, Université Cadi Ayyad, Centre Hospitalier Universitaire Mohammed VI, Marrakech, Maroc

**Keywords:** Traumatisme rénal, traumatisme rénal de haut grade, Scanner spiralé, kidney trauma, high grade kidney trauma, spiral CT

## Abstract

Les traumatismes graves du rein de grade III, IV et V selon la classification de l'Amercan Society for Surgery For Trauma (ASST) sont plus rares et se retrouvent dans 5% des cas en moyenne. Leur prise en charge est souvent délicate, nécessitant alors des centres expérimentés dotés de moyen adéquats d'imagerie (scanner spiralé). Cependant, durant ces dernières années, la prise en charge de ces traumatismes a évolué vers une attitude de moins en moins chirurgicale grâce à l’évolution des techniques de la radiologie interventionnelle, de l'endourologie et des moyens de surveillance aux urgences et de réanimation. L'objectif de cette étude est d’évaluer notre expérience dans la prise en charge des traumatismes rénaux de haut grade. Notre étude rétrospective porte sur 25 cas de traumatismes grave du rein de grade III, IV et V selon la classification de l'ASST, colligés entre Janvier 2002 et Juin 2009 au service d'urologie du centre Hospitalier Universitaire Mohammed VI, Université Cadi Ayyad de Marrakech, Maroc. Nous avons étudié les données épidémiologiques, les signes cliniques et biologiques à l'admission (état de choc hémorragique, taux d'hémoglobine), les données radiologiques (échographie et scanner), les lésions associées, la prise en charge thérapeutique et les complications. L’âge moyen de nos patients était de 24,9 ans 15 et 58 ans, avec une prédominance masculine (sex-ratio = 7, 3). Le rein droit était intéressé dans 15 cas (60%). Le traumatisme rénal était fermé dans 15 cas, et ouvert par arme blanche dans 10 cas. Huit patients se sont présentés en état de choc hémorragique (32%). Une anémie inférieur à 10g /100ml a été observée dans 10 cas (40%). L'uroscanner fait systématiquement à l'admission a retrouvé un grade III (10 cas), grade IV (13 cas) et grade V (2 cas). La prise en charge a consisté en une exploration chirurgicale avec néphrectomie chez 2 cas de Grade IV pour une instabilité hémodynamique. Une surveillance active clinique, biologique, et radiologique a été préconisée dans 23 cas (92%). Le scanner de contrôle fait à J7, a objectivé une stabilisation des lésions dans 17 cas et la constitution d'un urinome dans 2 cas drainé par sonde double J. Une néphrectomie d'hémostase était nécessaire dans 4 cas de grade IV (3 cas) et de grade V (1 cas). Un patient est décédé à J2 d'un traumatisme ouvert grade IV suite à une hémorragie foudroyante. La durée moyenne d'hospitalisation était de 17 jours (6-75 jours). A travers notre étude, on conforte l'attitude conservatrice recommandée actuellement dans la prise en charge de la plupart des traumatismes graves du rein en l'absence d'une instabilité hémodynamique, grâce aux mesures de réanimation correcte, une surveillance rapprochée du patient et le recours aux moyens endoscopiques de drainage des voies excrétrices. Grace à cette attitude la plupart de nos patients (76%) on conservé leur unités rénales, extrêmement précieuses en particulier pour les patients sujets à la dégradation ultérieure de leur fonction rénale, leur permettant ainsi d’éviter de tomber dans l'hémodialyse ou de rentrer dans des programmes de greffe rénale.

## Introduction

Les traumatismes graves du rein de grade III, IV et V selon la classification de l'ASST sont plus rares et se retrouvent dans 5% des cas en moyenne. Les indications d'imageries sont aujourd'hui bien codifiées et le scanner spiralé représente l'examen de référence. Durant ces dernières années, la prise en charge de ces traumatismes a évolué vers une attitude de moins en moins chirurgicale grâce à l’évolution des techniques de la radiologie interventionnelle, de l'endourologie et des moyens d'accueils aux urgences et de réanimation. Le but de cette étude était d’évaluer notre expérience en matière de prise en charge des traumatismes graves du rein.

## Méthodes

De Janvier 2002 au juin 2009, parmi 52 traumatismes rénaux colligés au service d'urologie du centre hospitalier universitaire Mohammed VI de Marrakech, le diagnostic de traumatisme rénal grave (selon la classification de l'American Association for the Surgery of Trauma (ASST) a été porté chez 25 patients. Nous avons étudié les données épidémiologiques, les signes cliniques et biologiques à l'admission (état de choc hémorragique, taux d'hémoglobine), les données radiologiques (échographie et scanner), les lésions associées, la prise en charge thérapeutique et les complications. Un uroscanner de contrôle a été systématiquement réalisé entre J5 et J7 du traumatisme.

## Résultats

**Figure 1:**
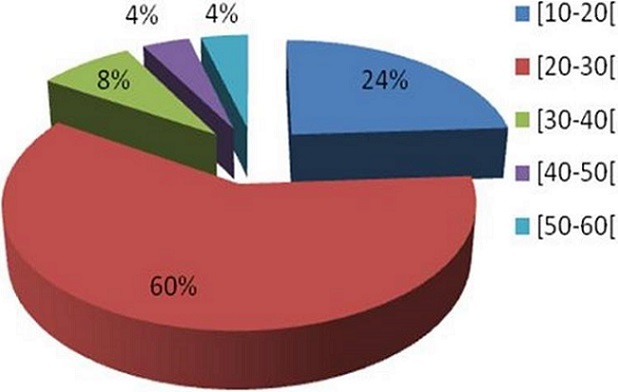
Répartition selon l’âge

**Figure 2:**
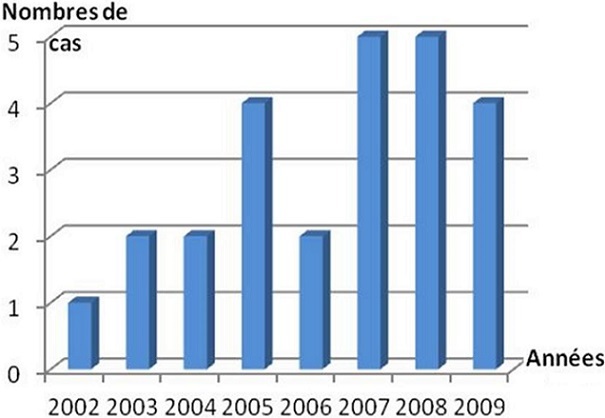
Répartition des patients selon l'année de survenue du traumatisme renal

**Figure 3:**
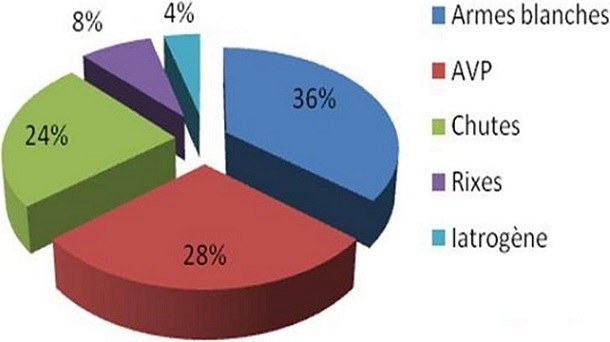
Étiologies des traumatismes rénaux graves

L’âge moyen de nos patients était de 24,9 ans (15-58), avec une prédominance masculine (sex-ratio = 7/ 3), (Figure 1). La répartition des patients selon l'année de survenue des traumatismes était très disparate avec un maximum de patients atteints en 2007 et en 2008 (Figure 2). Le rein droit était intéressé dans 15 cas (60%). Le traumatisme rénal était fermé dans 15 cas, et ouvert (pénétrant) par arme blanche dans 10 cas. Les étiologies du traumatisme rénal grave étaient dominées par les agressions par arme blanche dans 9 cas (36%), suivies des accidents de la voie publique dans 7 cas (28%), puis les chutes d'un lieu élevé dans 6 cas (24%), (Figure 3). Cependant, trois patients ont eu une uropathie sous jacente, un rein en fer à cheval dans deux cas et une lithiase rénale bilatérale dans un cas. A leur admission tous les patients présentaient des douleurs lombaires. Une hématurie macroscopique était associée dans 18 cas (72%). Huit patients (32%) se sont présentés en état de choc hémorragique et une transfusion sanguine était nécessaire dans 10 cas (40%), dont 7 cas (28%) réalisée dès l'admission aux urgences. Une anémie inférieur à 10g /100ml a été observée dans 10 cas (40%) (Tableau 1).

**Tableau 1:** Signes cliniques

signes cliniques	Nombre de cas	Fréquence%
Etat de choc	8	32
Hématurie	18	72
Lombalgies	25	100
Défense lombaire	17	68
Empâtement de FL	8	32

Les lésions associées aux traumatismes rénaux sont représentées dans le Tableau 2. L’échographie abdominale a retrouvé une contusion rénale dans 14 cas (56%), un hématome périrénal dans 14 cas (56%), un épanchement péritonéal dans 11 cas (44%), une fracture rénale unique dans 5 cas (20%) et multiple dans 2 cas (8%) et la présence de lésions d'autres viscères dans 4 cas (16%) avec fracture splénique dans 2 cas, contusion hépatique dans un cas et plaie diaphragmatique dans un seul cas. La répartition des grades III, IV et V après réalisation de l'uroscanner était respectivement de 10, 13 et 2 cas.

**Tableau 2:** Lésions associées

Organes	Nombre de cas
Fracture splénique	2
Contusion hépatique	1
Fracture du bassin	2
Traumatisme du thorax	2
Traumatisme crânien	1
Traumatisme de la face Plaie diaphragmatique	1 1

Une surveillance active clinique, biologique, et radiologique a été préconisée pour 23 patients (92%). Le scanner de contrôle était réalisé à J7 du traumatisme chez la plupart des patients. Il avait objectivé une stabilisation des lésions chez 17 patients et la constitution d'un urinome chez 2 patents drainés au moyen d'une sonde double J. Par ailleurs, une néphrectomie d'hémostase était nécessaire pour 6 patients de grade IV (3 cas) et de grade V (3 cas). Un patient est décédé d'un traumatisme ouvert qui était stable jusqu’à J2. La durée moyenne d'hospitalisation était de 17 jours (6 - 75 jours).

Tous les patients ont été suivis en consultation après leur sortie avec une médiane de 22 mois (extrêmes d'un mois à six ans). La surveillance comportait un examen clinique complet avec surveillance de la tension artérielle, dosage de la créatinine sérique et réalisation d'une échographie et/ou d'un uro-scanner de contrôle. Il n'a pas été constaté d'hypertension artérielle ou d'insuffisance rénale chez les patients surveillés ou opérés. 1 cas de mutité rénale sur l'euro-scanner de contrôle à 3 mois a été retrouvé.

## Discussion

Les traumatismes rénaux concernent l'homme jeune de préférence entre 10 et 40 ans, avec généralement une nette prédominance masculine [1], comme c'est le cas dans notre étude. Plusieurs équipes ont retrouvé une atteinte préférentielle du rein gauche [1] en cas de traumatisme rénal isolé, pour d'autre c'est le rein droit qu’était plus intéressé [2, 3], l'atteinte rénale bilatérale restant exceptionnelle. Ces traumatismes sont en rapport avec un accident de la voie publique dans 50 à 70% des cas (les piétons étant les plus exposés), une chute dans 15% des cas et un accident de sport dans 10% des cas [1].

Du point de vue clinique dans les traumatismes rénaux majeurs, les douleurs lombaires sont le plus souvent associées à une hématurie macroscopique ou à des signes de choc [4, 5]. Ces signes cliniques, suggérant la présence d'un traumatisme rénal grave, doivent conduire à la réalisation d'un bilan radiologique immédiat chez des patients stables hémodynamiquement initialement ou après réanimation hydroélectrolytique. L’échographie abdominopelvienne couplée au doppler couleur est souvent utilisée comme examen de débrouillage ("screening") en salle de déchoquage aux urgences et permet la réalisation d'un bilan lésionnel rapidement chez les patients instables hémodynamiquement. Elle est performante pour les lésions abdominales associées, notamment hépatiques et spléniques, mais pas pour celles du pancréas ou de l'intestin grêle [2, 6]. Chez les patients ayant des paramètres hémodynamiques stables, le scanner abdominopelvien avec des coupes sans injection, puis avec injection précoce et tardive reste l'examen clé pour étudier l’état du rein [2], rechercher les lésions associées et stadifier la lésion rénale. Il permet, d'une part, de localiser les lésions parenchymateuses, de rechercher une extravasation du produit de contraste, et de localiser les zones avasculaires. D'autre part, il sert à quantifier l'importance de l'hématome rétropéritonéal, à vérifier l’état du rein controlatéral (morphologie et fonction) et à rechercher l'existence ou non de lésions pédiculaires et des viscères intrapéritonéaux [1, 7, 8]. L'artériographie est généralement réalisée lorsque des lésions de grade 3, 4 ou 5 suivant la classification de l'ASST sont retrouvées au scanner [7]. Elle est réalisée chez des patients stables hémodynamiquement initialement ou stabilisés après réanimation. Les patients en état de choc malgré la réanimation (par rupture complète de l'artère ou du pédicule rénal), ainsi que les lésions de la veine rénale restent des indications à une exploration chirurgicale en urgence [7]. L'artériographie permet dans certains cas le traitement de lésions vasculaires par embolisation (généralement réalisée dans les lésions de grade 4 ou 5) durant le même temps opératoire (utilisation de colles biologiques ou coils). Les techniques d'embolisation sont réalisées en cas de lésions minimes de l'artère rénale ou des ses branches (cathetérisme sélectif), le but étant de stopper un saignement actif et d’éviter un geste chirurgical avec un risque de perte d'unité rénale [7]. Ces techniques d'embolisation sont aussi utilisées en cas de récidive hémorragique chez des patients non opérés ou en cas de fistule artérioveineuse ou de pseudo-anévrysme [7]. Elles sont une alternative aux techniques chirurgicales. Néanmoins, le recours à la chirurgie s'avère parfois nécessaire en cas d’échec de l'embolisation ou d'une reprise hémorragique différée [7]. Face à des lésions graves chez des patients instables hémodynamiquement malgré une réanimation correcte avec transfusion sanguine, la plupart des équipes s'accordent sur la nécessité d'une exploration chirurgicale en urgence qu'il y ait ou non des lésions viscérales associées [8, 9]. Cela se traduit dans la majorité des cas par une néphrectomie totale à visée d'hémostase du fait de lésions irréparables ou de l'urgence vitale [8].

Cependant, lorsque la réparation des lésions est possible, le taux de néphrectomie varie de 14 à 26%, grâce au contrôle premier des vaisseaux selon le principe décrit par McANINCH [10]. Pour les patients stables initialement ou après réanimation, et depuis 1995, nous avons opté pour une abstention chirurgicale vis à vis de ces traumatismes, selon une attitude consensuelle de nos jours [8, 9]. Une fistule urinaire, un urinome, ou une collection abcédée peuvent être traités par un geste endoscopique rétrograde (montée de sonde urétérale simple ou de sonde double J) ou par un drainage par voie percutanée évitant ainsi un abord chirurgical [8, 9]. Selon certains auteurs, une geste chirurgical est nécessaire dans seulement 10% des cas [1]. Concernant les patients opérés pour une lésion intrapéritonéale associée, l'exploration de la loge rénale ne doit pas être systématique [8, 9]. En l'absence de signes de gravité (hématome rétropéritonéal expansif ou pulsatile) [11], il n'y a pas lieu d'explorer la loge rénale, le bilan radiologique rénal pouvant être réalisé en postopératoire si cela n'a pas été le cas en préopératoire. Les lésions de grade 5 sont rares et représentent 1 à 4% des lésions dans les traumatismes fermés du rein [12]. Les lésions du pédicule rénal (artères, veines ou les deux) peuvent toucher soit le vaisseau principal, soit les branches de division et sont classées en avulsions, lacérations, ou occlusions (un cas dans notre série) [12]. Une lésion de l'artère, de la veine rénale ou de leurs branches s'observent dans 25% des cas dans les traumatismes rénaux graves nécessitant une exploration chirurgicale [11].

Le taux de néphrectomie totale en cas de lésion de la veine rénale varie entre 25 et 55% selon les séries, et en cas d'atteinte de l'artère rénale de 70 à 94% pour les traumatismes fermés [1]. Les lésions veineuses sont particulièrement difficiles à détecter (même au scanner) et à réparer. Le danger en cas de méconnaissance de ce type de lésion est une reprise hémorragique massive survenant généralement entre le 5ème et le 10^ème^ jour, une fois le caillot lysé (l'effet de tamponnade initiale réalisé par l'hématome périrénal ayant disparu) [1]. L'atteinte de la veine rénale, notamment sa section complète, est rarement réparable et nécessite souvent une néphrectomie du fait du saignement persistant. Cependant une ligature proximale de la veine rénale gauche n'entraîne pas forcément une néphrectomie totale, du fait de la présence du réseau de drainage collatéral réalisé par la veine gonadique, les veines lombaires et surrénaliennes [1, 12]. Concernant les lésions artérielles, lorsqu'une revascularisation est réalisée, elle fait appel aux différents procédés de réparation vasculaire (résection anastomose termino-terminale, greffon veineux ou artériel et autotransplantation). Dans la littérature, le succès à long terme de la revascularisation varie suivant les séries de 28,5% pour MAGGIO [13], 66,6% pour SMITH [14], 20 à 75% pour PIÉCHAUD [3]. Le succès de la revascularisation est lié à la durée d'ischémie chaude. Pour MAGGIO [13], les chances de succès de la revascularisation passe de 80% à 57% entre la 12ème et la 18ème heure. Pour EL KHADER [15], ce délai doit être inférieur à 4 heures, 12 heures pour SMITH [14] et 16 heures pour PIÉCHAUD [3]. Toutefois, il n'existe pas de consensus sur le délai “idéal” de revascularisation. Face à ce type de lésions, les chances de succès du geste de revascularisation dépendent de l'expérience du chirurgien mais également de la disponibilité d'un chirurgien vasculaire. Cependant, la réparation d'une lésion de l'artère rénale n'est pas toujours la garantie d'une restauration de la fonction rénale du rein lésé [7]. Concernant les occlusions ou thromboses artérielles; pour certains auteurs [1] un geste de revascularisation doit être tenté (temps d'ischémie chaude inférieur à 5 heures); pour d'autres [7, 12] si le temps d'ischémie est supérieur à 5 heures, si le patient est stable et la fonction rénale normale, le malade est à surveiller. En cas d’échec de la revascularisation, une néphrectomie totale doit être envisagée si la fonction du rein controlatéral est normale afin de diminuer le risque d'une néphrectomie retardée en raison du risque de saignement, d'infection, ou d'hypertension artérielle (l'HTA varie de 28 à 57% chez les patients ayant un traitement conservateur et se développe dans les jours ou les années qui suivent le traumatisme) [12].

## Conclusion

A travers notre étude, on conforte l'attitude conservatrice recommandée actuellement dans la prise en charge de la plupart des traumatismes graves du rein en l'absence d'une instabilité hémodynamique, grâce aux mesures de réanimation correcte, une surveillance rapproché du patient et le recours aux moyens endoscopiques de drainage des voies excrétrices. Grace à cette attitude la plupart de nos patients (76%) ont conservé leurs unités rénales, qui sont extrêmement précieuses en particulier pour les patients sujets à la dégradation ultérieures de leur fonction rénale, leur permettant ainsi d’éviter l'insuffisance rénale terminale et de préserver leur capital néphronique précieux.
